# The proportion of CD161 on CD56^+^ NK cells in peripheral circulation associates with clinical features and disease activity of primary Sjögren's syndrome

**DOI:** 10.1002/iid3.1244

**Published:** 2024-04-05

**Authors:** Ping Zhao, Yanhong Yang, Saizhe Song, Wei Cheng, Cheng Peng, Xin Chang, Jian Wu, Cuiping Liu

**Affiliations:** ^1^ Department of Rheumatology and Clinical Immunology The First Affiliated Hospital of Bengbu Medical University Bengbu China; ^2^ Anhui Province Key Laboratory of Basic and Translational Research of Inflammation‐related Diseases Bengbu China; ^3^ Jiangsu Institute of Clinical Immunology & Jiangsu Key Laboratory of Clinical Immunology The First Affiliated Hospital of Soochow University Suzhou China; ^4^ Department of Obstetrics and Gynecology The Second Affiliated Hospital of Soochow University Suzhou China; ^5^ Department of Rheumatology The First Affiliated Hospital of Soochow University Suzhou China; ^6^ Department of Dermatology Affiliated Changshu Hospital of NanTong University Suzhou, China

**Keywords:** CD161, disease activity, NK cells, primary Sjögren's syndrome

## Abstract

**Objectives:**

The purpose of this study was to examine the proportion of CD161 on CD56^+^ natural killer (NK) cells in peripheral blood of primary Sjögren's syndrome (pSS) and investigate its clinical relevance of pSS.

**Methods:**

The proportion of CD56^+^ NK cells and CD161 on CD56^+^ NK cells was detected by flow cytometry in 31 pSS patients and 29 healthy controls (HCs). The correlations between the proportion of CD161^+^CD56^+^ NK cells and clinical features and disease activity of pSS were further analyzed. Meanwhile, we drew the receiver operating characteristic curve to evaluate the diagnostic value of CD161^+^CD56^+^ NK cells in pSS. In addition, we evaluated the differences in the effects of CD161^+^ cells and CD161^−^ cells in peripheral blood on the function of CD56^+^ NK cells in 5 pSS patients.

**Results:**

The proportion of CD56^+^ NK cells and CD161^+^CD56^+^ NK cells decreased markedly in pSS patients compared to HCs. The correlation analysis showed that the proportion of CD161^+^CD56^+^ NK cells negatively correlated with white blood cells, Immunoglobulin A (IgA), IgM, IgG, European League Against Rheumatism Sjogren's Syndrome Patient Reported Index and European League Against Rheumatism Sjogren's Syndrome Disease Activity Index, and positively correlated with complement C4. The proportion of CD161^+^CD56^+^ NK cells in pSS patients with decayed tooth, fatigue, arthralgia, skin involvement, primary biliary cirrhosis, interstitial lung disease, anti‐SSA/Ro60 positive, anti‐SSB positive and high IgG was lower than that in negative patients. Furthermore, compared with inactive patients, the proportion of CD161^+^CD56^+^ NK cells decreased obviously in active patients. The area under the curve was 0.7375 (*p* = .0016), the results indicated that CD161^+^CD56^+^ NK cells had certain diagnostic values for pSS. In addition, the proportion of CD86, HLA‐DR, Ki67, FasL, TNF‐α, and IFN‐γ on CD161^+^CD56^+^ NK cells was lower than that on CD161^−^CD56^+^ NK cells in the peripheral blood of pSS patients.

**Conclusion:**

This study suggested that the proportion of CD56^+^ NK cells and CD161^+^CD56^+^ NK cells decreased significantly in pSS patients, and the proportion of CD161^+^CD56^+^ NK cells negatively associated with the clinical features and disease activity of pSS patients. CD161 expression inhibited the function of CD56^+^ NK cells in peripheral blood of pSS patients. The CD161^+^CD56^+^ NK cells may present as a potential target for therapy and a biomarker of disease activity in pSS.

## 1 INTRODUCTION

Primary Sjögren's syndrome (pSS) is a complex autoimmune disease, which is characterized by the infiltration of lymphocytes from exocrine glands, leading to the dysfunction of gland secretion, and then a series of symptoms of gland injuries, such as dry mouth, dry eye and swelling of parotid gland, as well as extra‐glandular manifestations such as arthralgia, interstitial lung disease (ILD), renal tubular acidosis and primary biliary cirrhosis (PBC), and part of patients have systemic manifestations, such as fatigue, fever, and weight loss.^1^, ^2^ Until now, the pathogenesis of pSS has not been clarified. Generally speaking, the pathogenesis of pSS is related to environmental factors, genetic susceptibility, and immune disorder. It is well known that pSS is mainly mediated by T cells and B cells, but innate immune cells such as NK cells also play an important role in the pathogenesis of pSS.^3^ Recent studies have found that the expression of NK cells in peripheral blood or salivary glands of pSS patients or model mice was abnormal, which was related to the activity and severity of the disease.^4^, ^5^, ^6^

CD161 is one of the members of human NKRP1 subfamily, which is called NK cell receptor protein 1A (NKR‐P1A) or killer cell lectin‐like receptor subfamily B member 1 (KLRB1), and it is also a type II transmembrane C‐type lectin glycoprotein receptor, which is called C‐type lectin domain family 5 member B (CLEC5B).^7^ CD161 is mostly expressed in NK cells and T cell subsets such as CD4^+^ T cells, CD8 αβT cells, γδT cells, and NKT cells.^8^ In humans, the ligand of CD161 is called lectin‐like transcript 1 (LLT1), which is mainly expressed on activated monocytes and B cells, and interacts with CD161 to inhibit the activation of NK cells and inhibit cytotoxic function and cytokine secretion mediated by NK cells.^9^, ^10^

At present, the research on CD161 in pSS patients is limited to T cells and the subsets of T cells,^11^, ^12^ and there is no relevant study on the clinical relevance of CD161 expression on NK cells in pSS patients. In this study, we detected the proportion of CD161 in peripheral blood on CD56^+^ NK cells of pSS patients by flow cytometry and analyzed the clinical correlation between the proportion of CD161^+^CD56^+^ NK cells and disease activity in pSS. This research aims to provide new ideas and therapeutic targets for the diagnosis and treatment of pSS.

## 2 MATERIALS AND METHODS

### 2.1 Patients and healthy controls

A total of 31 pSS patients were enrolled from the Rheumatology and Immunology Department, The First Affiliated Hospital of Soochow University in the study. All patients were diagnosed according to the classification standard of primary Sjögren's syndrome of the American College of Rheumatology (ACR)/European League Against Rheumatism (EULAR) in 2016,^13^ and those with malignant tumor, chronic hepatitis C (HCV), human immunodeficiency virus infection (HIV), sarcoidosis, amyloidosis, graft versus host disease (GVHD), IgG4‐related diseases, and other rheumatic diseases were excluded. We also recruited 29 individuals (age‐ and gender‐matched) without autoimmune diseases, cancer, and infectious diseases from the physical examination center as healthy controls (HCs). This research was approved by the Ethics Committee of the First Affiliated Hospital of Soochow University in 2020 (Ethical No. 2020105). All participants signed the informed consent.

### 2.2 Data collection

We recorded the detailed clinical manifestations of these pSS patients, such as dry mouth, dry eye, decayed tooth, fatigue, arthralgia, Raynaud's phenomenon, skin involvement, gland involvement, fever, muscle involvement, renal tubular acidosis, interstitial lung disease (ILD) and primary biliary cirrhosis (PBC), and so forth. At the same time, the laboratory data such as white blood cell count, red blood cell count, platelet count, erythrocyte sedimentation rate (ESR), C‐reactive protein (CRP), rheumatoid factor (RF), serum globulin, immunoglobulin (IgG, IgA, IgM), complement (C3, C4) and auto‐antibodies (anti‐SSA/Ro52, anti‐SSA/Ro60, anti‐SSB and anti‐centromere) were collected. The blood samples for the above tests were fasting blood from pSS patients in the morning. According to the above clinical manifestations and laboratory parameters, the European League Against Rheumatism Sjögren's Syndrome Disease Activity Index (ESSDAI) and European League Against Rheumatism Sjögren's Syndrome Patient Reported (ESSPRI) of patients were evaluated.^14^ ESSPRI is the patient's subjective score, which is the average of the three points, including dryness, pain, and fatigue, and each evaluation score range is 0 to 10. ESSDAI is the pSS disease activity index, which is calculated according to the patient's clinical manifestations, laboratory parameters, and corresponding weight coefficient. At the same time, general information about HCs such as gender and age were collected.

### 2.3 Flow cytometric analysis

#### 2.3.1 Human monoclonal antibodies were used in this study

We used the following human fluorescent monoclonal antibodies markers: anti‐CD56‐FITC (Bio‐legend), anti‐CD161‐PE (Bio‐legend), anti‐CD56‐BV421 (Bio‐legend), anti‐CD86‐FITC (Bio‐legend), anti‐GranzymeB‐PE/Cyanine7 (Bio‐legend), anti‐perforin‐APC/Cy7(Bio‐legend), anti‐Ki67‐AlexaFluor™700 (Bio‐legend), anti‐FasL‐PerCP/Cy5.5 (Bio‐legend), anti‐HLA‐DR‐FITC (Bio‐legend), anti‐TNF‐α‐PE/Cyanine7 (Bio‐legend), and anti‐IFN‐γ‐APC/Cy7 (Bio‐legend). The isotype control antibodies used in our study were as follows: Mouse IgG 1κ‐FITC (Bio‐legend), Mouse IgG1κ‐PE (Bio‐legend), Mouse IgG2bκ‐BV421 (Bio‐legend), Mouse IgG1κ‐PE/Cyanine7 (Bio‐legend), Mouse IgG2ακ‐Alexa Fluor™700 (Bio‐legend), Mouse IgG1κ‐PerCP/Cy5.5 (Bio‐legend), and Mouse IgG2α κ‐APC/Cy7 (Bio‐legend).

#### 2.3.2 Surface staining of CD161

Fresh venous blood of pSS patients and HCs on an empty stomach were collected with heparinized anti‐coagulation tubes. According to the instructions of the manufacturer, 50 μL whole blood samples were stained with human CD56‐FITC antibody and human CD161‐PE antibody, and incubated at 4°C in the dark for 30 min. Then, 200 μL/test of erythrolysin (1×) was added into all sample tubes, and all samples were vibrated by an oscillator and placed in a thermostat at 37°C to fully lyse red blood cells. Then, 2 mL/test sheath fluid was added into all samples for washing and centrifugation (1200 rpm, 5 min), and the waste liquid was carefully dumped (take and put the test tubes gently to avoid vibration!). Next, 0.5 mL/test of sheath fluid was added to resuspend white blood cells to obtain leukocyte suspension for detection.

#### 2.3.3 Intracellular staining of cytokines

Peripheral blood mononuclear cells (PBMCs) of pSS patients were isolated by Ficoll‐Hypaque density gradient centrifugation. Adding phorbol 12‐myristate 13‐acetate (PMA) and ionomycin into PBMCs which were incubated with RPMI1640 culture medium, stimulating in a 37% CO_2_ incubator for 1 h, adding protein transport inhibitor Golgistop, and then reacting in a refrigerator at 4°C for overnight. The next day, extracting PBMCs and adding membrane surface human monoclonal antibodies CD56‐BV421, CD161‐PE, CD86‐FITC, FasL‐PerCP/Cy5.5, and HLA‐DR‐FITC, and reacting in a refrigerator at 4°C for 30 min. Then, adding fixed and permeabilized buffers and human monoclonal antibodies Granzyme B‐PE/Cyanine7, perforin‐APC/Cy7, Ki67‐AlexaFluor™700, TNF‐α‐PE/Cyanine7, and IFN‐γ‐APC/Cy7, reacting in a refrigerator at 4°C for 30 min. The flow cytometric analysis was processed by flow cytometry (FC500; Beckman Coulter), and the data results were analyzed by FlowJo 10.8.1 software.

### 2.4 Statistical analysis

All data were collected using GraphPad Prism (version 8.0.2) software for statistical analysis and graphic presentations. Flowjo software (version 10.8.1) was used to analyze the flow cytology data. The normality of the data was tested by the Shapiro‐Wilk test (*n* < 50). Normally distributed data were expressed as mean ± standard deviation, and non‐normally distributed data were represented by the median (minimum number–maximum number). The Mann–Whitney *U* test was used for the data without normal distribution. The *T* test was used for normally distributed paired samples. The correlation analysis between two continuous variables that were non‐normally distributed was analyzed using Spearman's rank correlation. The statistical significance was determined as *p* < .05 (*p < .05, **p < .01, ****p < .0001).

## 3 RESULTS

### 3.1 Essential information of participants

A total of 31 pSS patients (29 females, two males) and 29 HCs (28 females, one male) were recruited in this study. There was no significant difference in age between pSS and HCs groups (47.13 ± 11.94 vs. 43.69 ± 8.79, *p* = .212). The mean disease duration of pSS patients was 46 months, ranging from 1 month to 122 months. The clinical manifestations and laboratory parameters of the enrolled participants are presented in Table 1.

**Table 1:** Clinical characteristics and laboratory parameters of included participants.

	pSS	HC
Number	31	29
Age (years)	47.13 ± 11.94	43.69 ± 8.79
Female, *n* (%)	29 (93.55%)	28 (96.55%)
Male, *n* (%)	2 (6.45%)	1 (3.45%)
Disease duration (months)	46 (1–122)	NA
Major clinical features		
Dry mouth, *n* (%)	24 (77.42%)	NA
Dry eye, *n* (%)	15 (48.39%)	NA
Decayed tooth, *n* (%)	11 (35.48%)	NA
Glandular swelling, *n* (%)	5 (16.13%)	NA
Raynaud's phenomenon, *n* (%)	3 (9.68%)	NA
Fatigue, *n* (%)	15 (48.39%)	NA
Weigh loss, *n* (%)	1 (3.23%)	NA
Arthralgia, *n* (%)	9 (29.03%)	NA
Skin involvement, *n* (%)	6 (19.35%)	NA
Primary biliary cirrhosis, *n* (%)	5 (16.13%)	NA
Renal involvement, *n* (%)	4 (12.90%)	NA
Interstitial lung disease, *n* (%)	11 (35.48%)	NA
Fever, *n* (%)	1 (3.23%)	NA
Muscle involvement, *n* (%)	1 (3.23%)	NA
Nervous system involvement, *n* (%)	1 (3.23%)	NA
High‐IgG	20 (64.52%)	NA
Leukocytopenia	5 (16.13%)	NA
Major laboratory features		
ESR (mm/h)	14.50 (2.00–62.00)	NA
CRP (mg/L)	1.76 (0.20–8.91)	NA
RF (IU/ml)	46.20 (20.00–777.00)	NA
WBC (×10^9^/L)	4.72 (2.79–11.78)	NA
Lymphocyte (×10^9^/L)	1.55 ± 0.60	NA
NC (×10^9^/L)	2.69 (1.44–8.74)	NA
RBC (×10^12^/L)	4.23 ± 0.38	NA
Hb (g/L）	126 ± 11.26	NA
Plt (×10^9^/L)	203.80 ± 49.31	NA
Serum globulin (g/L)	31.70 ± 6.13	NA
IgG (g/L)	17.84 ± 5.01	NA
IgA (g/L)	2.77 (1.40–10.60)	NA
IgM (g/L)	1.16 (0.41–7.35)	NA
C3 (g/L)	0.79 (0.62‐1.52)	NA
C4 (g/L)	0.19 ± 0.06	NA
Anti‐SSA/Ro52 (+), *n* (%)	28 (90.32%)	NA
Anti‐SSA/Ro60 (+), *n* (%)	24 (77.42%)	NA
Anti‐SSB (+), *n* (%)	20 (64.52%)	NA
Anti‐centromere (+), *n* (%)	3 (9.68%)	NA
ESSDAI	3.00 (1.00–8.00)	NA
ESSPRI	3.00 (1.33–5.00)	NA

Abbreviations: C3, Complement 3; C4, Complement 4; CRP, C‐reactive protein; ESR, erythrocyte sedimentation rate; ESSDAI, European League Against Rheumatism Sjogren's Syndrome Disease Activity Index; ESSPRI, European League Against Rheumatism Sjogren's Syndrome Patient Reported Index; Hb, hemoglobin; IgA, Immunoglobulin A; IgG, Immunoglobulin G; IgM, Immunoglobulin M; ILD, interstitial lung disease; NA, not applicable; NC, neutrophil cell; PBC, primary biliary cirrhosis; Plt, platelet; RBC, red blood cell; RF, rheumatoid factor; WBC, white blood cell.

### 3.2 The proportion of CD56^+^ NK cells and CD161^+^CD56^+^ NK cells decreased in pSS patients

**Figure 1:**
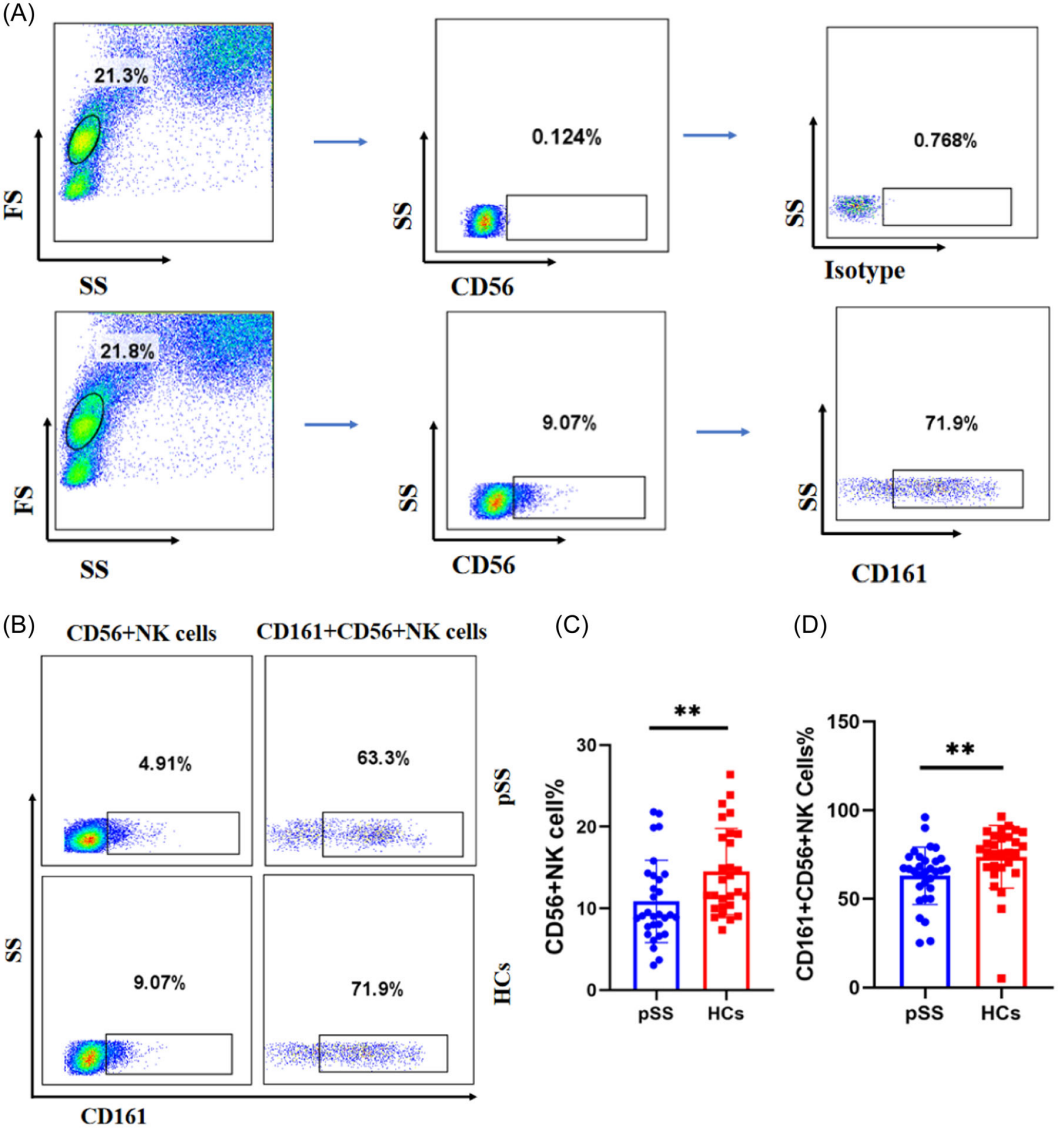
The proportion of CD56^+^ NK cells and CD161^+^CD56^+^ NK cells in primary Sjögren's syndrome (pSS) patients and healthy controls (HCs). (A) The gating strategies for CD56^+^ NK cells and CD161^+^CD56^+^ NK cells. (B) The representativeFACS plots. (C) The proportion of CD56^+^ NK cells decreased in pSS patients. (D) The proportion of CD161^+^CD56^+^ NK cells in pSS patients also decreased significantly (Mann–Whitney *U* test,**p < .01).

To evaluate the expression of CD56^+^ NK cells and CD161 on the surface of CD56^+^ NK cells in the peripheral blood of pSS patients and HCs, we used flow cytometry to detect the proportion of CD56^+^ NK cells and CD161^+^CD56^+^ NK cells. The results showed that the proportion of CD56^+^ NK CD56^+^ NK cells decreased markedly compared with HCs (9.16% [3.05%–21.80%] vs. 12.75% [7.36%–26.40%], *p* = .0052) (Figure 1C). Similarly, the proportion of CD161^+^CD56^+^ NK cells was also significantly lower than that in HCs (65.90% [25.15%–96.10%] vs. 76.10% [5.22%–96.40%], *p* = .0013) (Figure 1D). The gating strategies for CD56^+^ NK cells and CD161^+^CD56^+^ NK cells are represented in Figure 1A, and the representative FACS plots are shown in Figure 1B.

### 3.3 Difference of CD161^+^CD56^+^ NK cells proportion in pSS patients with different clinical manifestations

**Figure 2:**
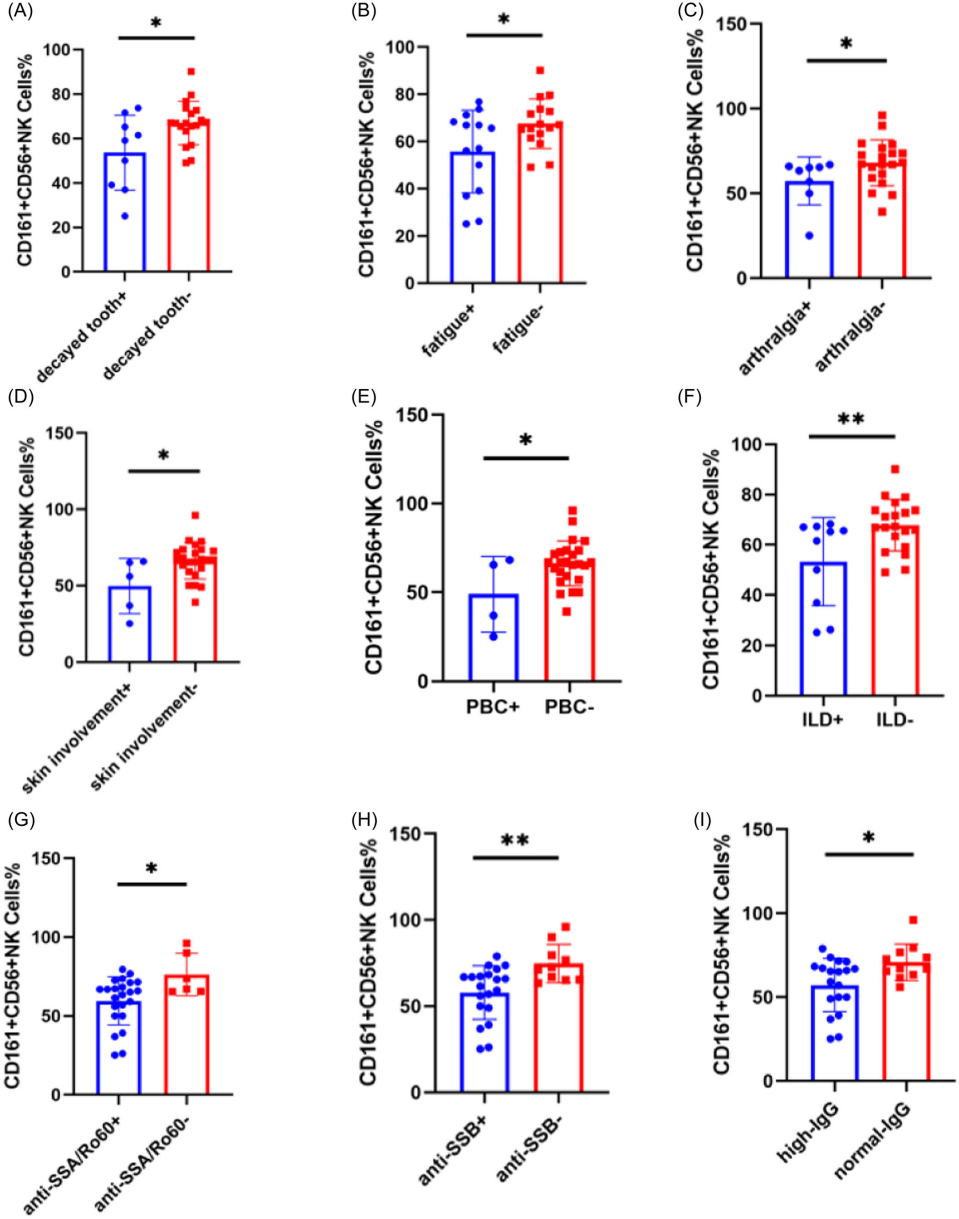
The proportion of CD161^+^CD56^+^ NK cells in different clinical manifestations, autoantibodies and IgG in primary Sjögren's syndrome (pSS) patients. The proportion of CD161^+^CD56^+^ NK cells decreased in pSS patients with decayed tooth (A), fatigue (B), arthralgia (C), skin involvement (D), primary biliary cirrhosis (PBC) (E), interstitial lung disease (ILD) (F), anti‐SSA/Ro60 positive (G), anti‐SSB positive (H), and high Immunoglobulin G (IgG) (I) (*T* test, *p < .05, **p < .01).

We compared the differences of CD161^+^CD56^+^ NK cells proportion in pSS patients with different clinical manifestations (Table 2), and it was found that the proportion of CD161^+^CD56^+^ NK cells with decayed tooth (53.60% ± 16.83% vs. 67.00% ± 9.81%, *p* = .013, Figure 2A), fatigue (55.71% ± 17.50% vs. 67.54% ± 10.49%, *p* = .030, Figure 2B), arthralgia (57.38% ± 14.24% vs. 69.69% ± 12.04%, *p* = .030, Figure 2C), skin‐involvement (49.84% ± 18.09% vs. 66.30% ± 11.94%, *p* = .016, Figure 2D), PBC (49.00% ± 21.32% vs. 66.31% ± 12.64%, *p* = .028, Figure 2E) and ILD (53.32% ± 17.57% vs. 67.78% ± 10.23%, *p* = .008, Figure 2F) in pSS patients notably decreased compared with pSS patients without above manifestations. We also analyzed the difference in the proportion of CD161^+^CD56^+^ NK cells between pSS patients with positive and negative auto‐antibodies. The results showed that the proportion of CD161^+^CD56^+^ NK cells in patients with anti‐SSA/Ro60 positive (59.59% ± 15.25% vs. 76.34% ± 13.47%, *p* = .021, Figure 2G) and anti‐SSB positive (57.96% ± 15.63% vs. 74.76 ± 11.06%, *p* = .005, Figure 2H) was significantly lower than that in patients with negative auto‐antibodies. Compared to pSS patients with normal IgG, the proportion of CD161^+^CD56^+^ NK cells in patients with high IgG obviously decreased (57.22% ± 15.95% vs. 70.85% ± 10.87%, *p* = .018, Figure 2I). However, there was no significant difference in the proportion of CD161^+^CD56^+^ NK cells between pSS patients with dry mouth, dry eye, gland involvement, leukopenia, and Raynaud's phenomenon and patients without the above clinical manifestations. The proportion of CD161^+^CD56^+^ NK cells only slightly decreased in anti‐SSA/Ro52 positive and anti‐centromere positive patients compared to anti‐SSA/Ro52 and anti‐centromere negative patients, but there was also no statistical difference.

**Table 2:** The proportion of CD161^+^CD56^+^ NK cells in different clinical manifestations, autoantibodies, and Immunoglobulin G (IgG) in primary Sjögren's syndrome (pSS) patients.

	With	Without	*t*	*p*
Dry mouth	62.15% ± 14.11%	68.50% ± 15.09%	0.970	0.341
Dry eye	64.73% ± 10.86%	66.74% ± 15.50%	0.407	0.687
Decayed tooth	53.60% ± 16.83%	67.00% ± 9.81%	2.671	0.013*
Fatigue	55.71% ± 17.50%	67.54% ± 10.49%	2.280	0.030*
Arthralgia	57.38% ± 14.24%	69.69% ± 12.04%	2.300	0.030*
Skin involvement	49.84% ± 18.09%	66.30% ± 11.94%	2.570	0.016*
Gland involvement	64.74% ± 11.84%	65.90% ± 13.58%	0.177	0.861
Leukocytopenia	67.25% ± 11.54%	64.18% ± 12.19%	0.470	0.643
Raynaud's phenomenon	67.04% ± 10.45%	64.05% ± 15.38%	0.326	0.747
PBC	49.00% ± 21.32%	66.31% ± 12.64%	2.316	0.028*
ILD	53.32% ± 17.57%	67.78% ± 10.23%	2.860	0.008**
High IgG	57.22% ± 15.95%	70.85% ± 10.87%	2.508	0.018*
Anti‐SSA/Ro52+	60.84% ± 15.21%	62.89% ± 6.00%	0.228	0.821
Anti‐SSA/Ro60+	59.59% ± 15.25%	76.34% ± 13.47%	2.445	0.021*
Anti‐SSB+	57.96% ± 15.63%	74.76% ± 11.06%	3.029	0.005**
Anti‐centromere+	59.94% ± 14.83%	70.72% ± 5.30%	1.233	0.228

Abbreviations: ILD, interstitial lung disease; PBC, primary biliary cirrhosis.

* *p* < .05

** *p* < .01

### 3.4 The proportion of CD161^+^CD56^+^ NK cells decreased significantly in active pSS patients

**Figure 3:**
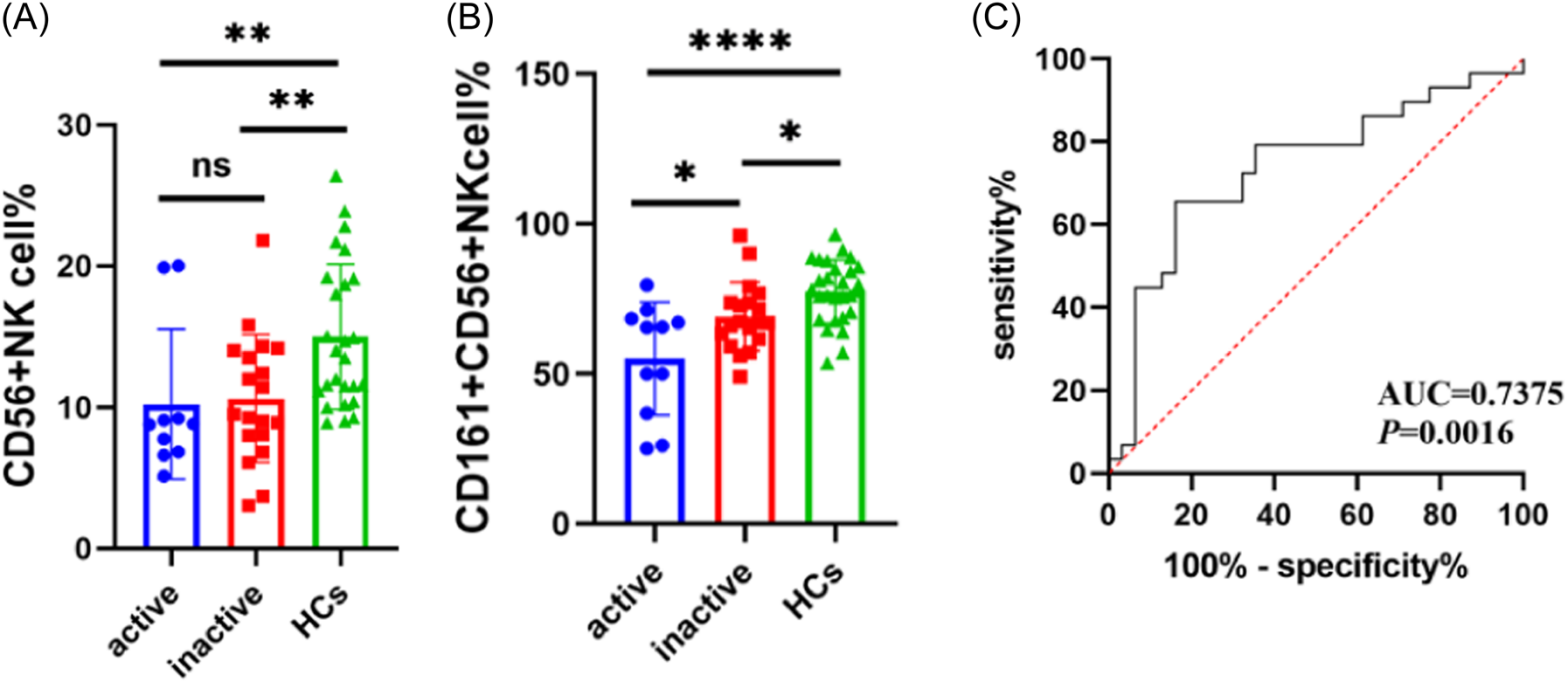
The proportion of CD56^+^ NK cells and CD161^+^ CD56^+^ NK cells in healthy controls (HCs), active and inactive primary Sjögren's syndrome (pSS) patients. (A) The proportion of CD56^+^ NK cells decreased in active and inactive pSS patients compared with HCs (Mann–Whitney *U* test, **p < .01). (B) The proportion of CD161^+^ CD56^+^ NK CD56^+^ NK cells was lower in active pSS than that in inactive pSS patients, and decreased in pSS patients significantly compared with HCs. (*T* test, *p < .05, ****p < .0001). (C) The receiver operating characteristic (ROC) curve of CD161^+^ CD56^+^ NK cells to predict the disease of pSS (AUC = 0.7375, *p* = .0016).

Based on the ESDDAI score, pSS patients with ESSDAI ≥ 5 were defined as active group, and ESSDAI < 5 were defined as inactive group.^15^ We analyzed the difference of proportion between CD56^+^ NK cells and CD161^+^CD56^+^ NK cells in active and inactive pSS patients and HCs, and we observed that CD56^+^ NK cells proportion decreased in active and inactive pSS patients compared with HCs (active vs. HCs: 8.81% [5.13%–20.00%] vs. 13.75% [8.90%–26.40%], *p* = .002; inactive vs. HCs: 9.50% [3.05%–21.80%] vs. 13.75% [8.90%–26.40%], *p* = .007) (Figure 3A), but there was no statistical difference between active and inactive pSS patients (8.81% [5.13%–20.00%] vs. 9.50% [3.05%–21.80%], *p* = .429) (Figure 3A). Regarding the difference of CD161^+^CD56^+^ NK cells proportion in active and inactive pSS patients and HCs, we found that not only CD161^+^CD56^+^ NK cells proportion in active and inactive pSS patients was obviously lower than that in HCs (active vs. HCs: 55.04% ± 18.73% vs. 77.38% ± 10.53%, *p* < .0001. inactive vs. HCs: 69.06% ± 11.39% vs.77.38% ± 10.53%, *p* = .014) (Figure 3B), but also the proportion of CD161^+^CD56^+^ NK cells in active pSS patients significantly decreased compared with inactive pSS patients (55.04% ± 18.73% vs. 69.06% ± 11.39%, *p* = .016) (Figure 3B).

### 3.5 CD161^+^CD56^+^ NK cells proportion was negatively correlated with disease activity in pSS patients

**Figure 4:**
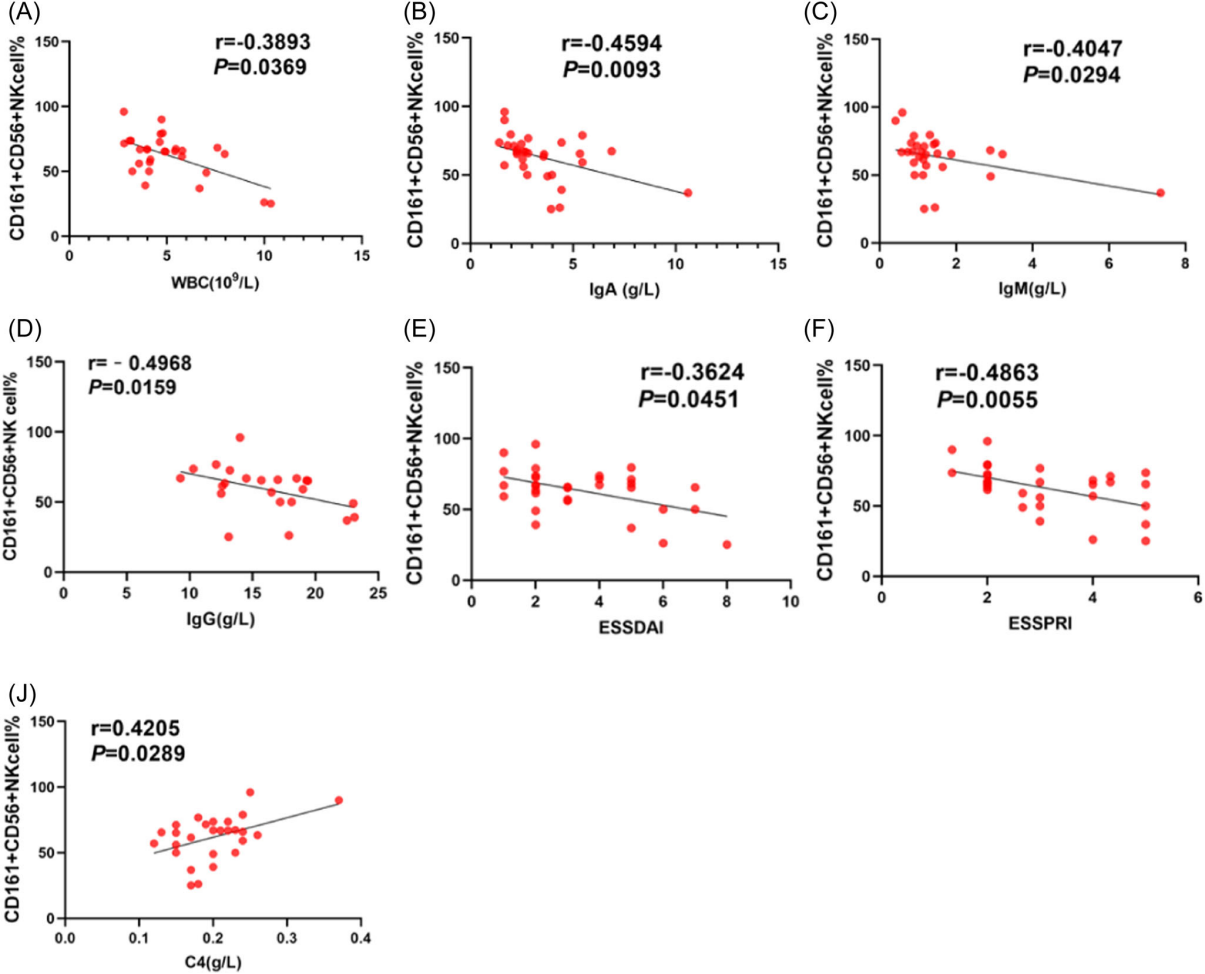
Correlations between CD161^+^CD56^+^ NK cells proportion and disease activity in primary Sjögren's syndrome (pSS) patients. Correlations between CD161^+^CD56^+^ NK cells proportion and white blood cell (WBC) (A), Immunoglobulin A (IgA) (B), Immunoglobulin M (IgM) (C), Immunoglobulin G (IgG) (D), European League Against Rheumatism Sjogren's Syndrome Disease Activity Index (ESSDAI) (E), European League Against Rheumatism Sjogren's Syndrome Patient Reported Index (ESSPRI) (F) and Complement 4 (C4) (J). Correlation analyses were used Spearman's rank correlation.

We subsequently analyzed the correlations between CD161^+^CD56^+^ NK cells proportion and laboratory features and disease activity index in pSS patients (Table 3). It was found that a positive correlation was verified between CD161^+^CD56^+^ NK cells proportion and C4 (*r* = .4205, *p* = .0289, Table 3, Figure 4J), and CD161^+^CD56^+^ NK cells proportion was negatively correlated with WBC (*r* = −0.3893, *p* = .0369, Table 3, Figure 4A), IgA (*r* = −0.4594, *p* = .0093, Table 3, Figure 4B), IgM (*r* = −0.4047, *p* = .0294, Table 3, Figure 4C), IgG (*r* = −0.4968, *p* = .0159, Table 3, Figure 4D), ESSDAI (*r* = −0.3624, *p* = .0451, Table 3, Figure 4E) and ESSPRI (*r* = −0.4863, *p* = .0055, Table 3, Figure 4F). However, there was no significant difference in correlations between CD161^+^CD56^+^ NK cells and ESR, serum globulin, and C3 (Table 3).

**Table 3:** Correlations between CD161^+^CD56^+^ NK cells proportion and laboratory features and disease activity index in primary Sjögren's syndrome (pSS) patients.

Laboratory parameters and disease activity index	CD161^+^CD56^+^ NK cells %	
	*r*	*p*
ESR	−0.3884	0.0607
WBC	−0.3893	0.0369
Serum globulin	−0.1095	0.5867
IgA	−0.4594	0.0093
IgM	−0.4047	0.0294
IgG	−0.4968	0.0159
C3	0.0934	0.6236
C4	0.4205	0.0289
ESSDAI	−0.3624	0.0451
ESSPRI	−0.4863	0.0055

Abbreviations: C3, Complement 3; C4, Complement 4; ESR, erythrocyte sedimentation rate; ESSDAI, European League Against Rheumatism Sjogren's Syndrome Disease Activity Index; ESSPRI, European League Against Rheumatism Sjogren's Syndrome Patient Reported Index; IgA, Immunoglobulin A; IgG, Immunoglobulin G; IgM, Immunoglobulin M; NK, natural killer; WBC, white blood cell.

### 3.6 The ROC curve of CD161^+^CD56^+^NK cells proportion to predict the occurrence of pSS

Combined with our above research results, we found that the proportion of CD161^+^CD56^+^ NK cells in pSS patients decreased significantly, and it was related to the clinical manifestations, laboratory parameters, and disease activities of pSS. Thus, we drew receiver operating characteristic (ROC) curve to evaluate the diagnostic value of CD161^+^CD56^+^NK cells for predicting the occurrence of pSS disease. The results showed that area under the curve (AUC) = 0.7375, *p* = .0016 (Figure 3C), and suggested that CD161^+^CD56^+^ NK cells had a certain diagnostic value for the occurrence of pSS.

### 3.7 CD161^+^ cells in peripheral blood of pSS patients inhibited the function of NK cells

**Figure 5:**
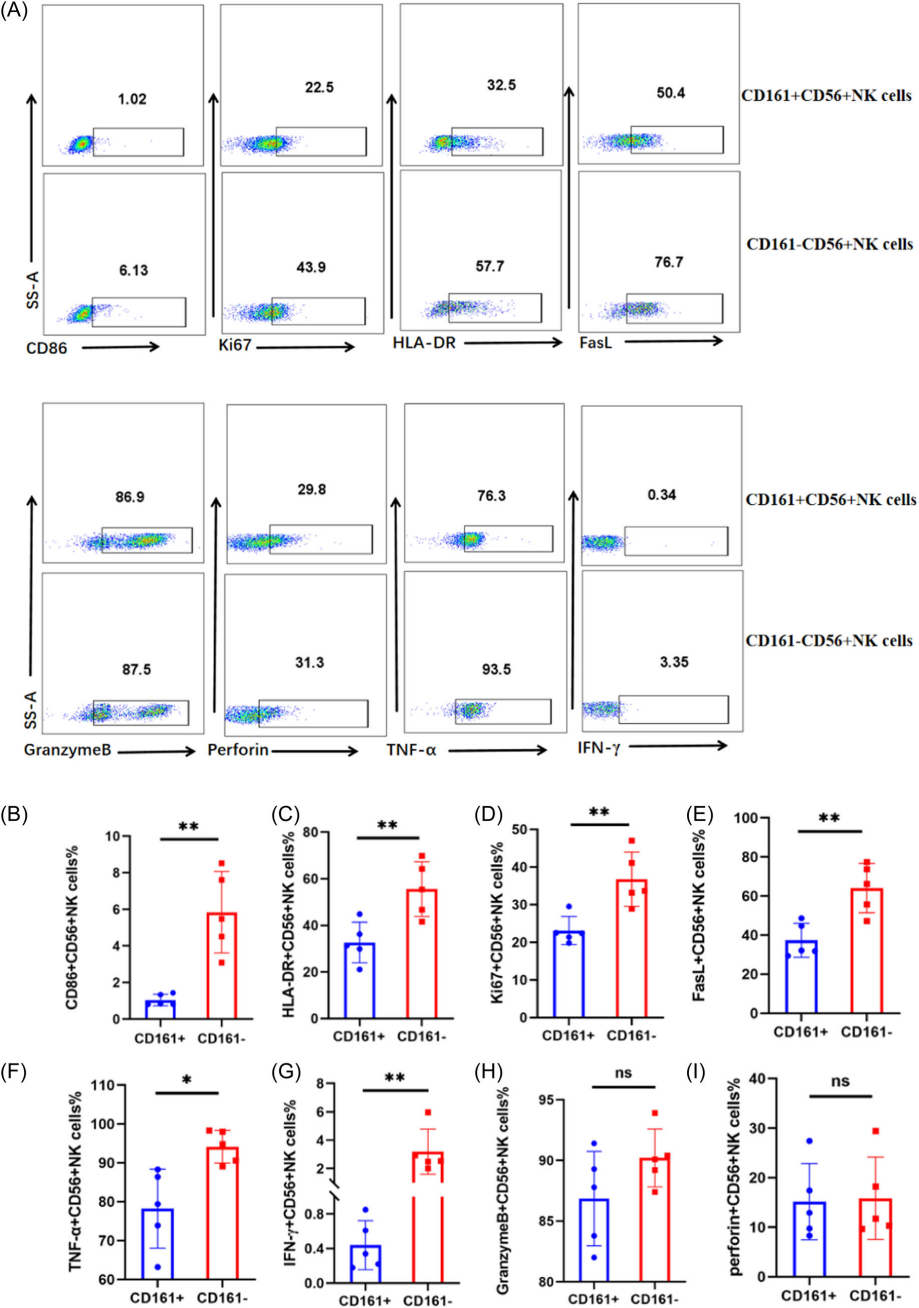
CD161^+^ cells in peripheral blood of primary Sjögren's syndrome (pSS) patients inhibited the function of CD56^+^ NK cells. *n* = 5. (A) The representative FACS plots. (B–G) The proportion of CD86 (B), HLA‐DR (C), Ki67 (D), FasL (E), TNF‐α (F) and IFN‐γ (G) on CD161^+^CD56^+^ NK cells was lower than that on CD161^−^ CD56^+^ NK cells in the peripheral blood of pSS patients (*p < .05, **p < .01). (H) The proportion of granzyme B on CD161^+^CD56^+^ NK cells was lower than that on CD161^−^ CD56^+^ NK cells in the peripheral blood of pSS patients, but there was no statistical difference (*p *＞ 0.05). (I) There was no statistical difference between the proportion of perforin on CD161^+^CD56^+^ NK cells and CD161^−^CD56^+^ NK cells (*p *＞ 0.05) (*T* test).

We further explored the effects of CD161 on the proliferation, activation, inducing apoptosis of target cells, and cytokine secretion of CD56^+^ NK cells. The gating strategies for CD86, granzyme B, perforin, Ki67, HLA‐DR, TNF‐α, interferon‐γ (IFN‐γ) and FasL on CD161^+^CD56^+^ NK cells and CD161^−^CD56^+^ NK cells are represented in Figure S1, and the representative FACS plots are shown in Figure 5A. In pSS patients, the proportion of surface active markers CD86 and HLA‐DR on CD161^+^CD56^+^ NK cells was lower than that on CD161­^−^CD56^+^ NK cells (CD86^+^ cells%: 1.05% ± 0.32% vs. 5.84% ± 2.22%, *p* = .0014, Figure 5B; HLA‐DR^+^ cells%: 32.66% ± 8.70% vs. 55.54% ± 11.73%, *p* = .0081, Figure 5C). Similarly, we observed that the expression proportion of proliferation marker Ki67 and pro‐inflammatory cytokines TNF‐α and IFN‐γ on CD161^+^CD56^+^ NK cells was significantly lower than that on CD161^−^CD56^+^ NK cells (Ki67^+^ cells%: 23.12% ± 3.73% vs. 36.78% ± 7.20%, *p* = .0055, Figure 5D; TNF‐α^+^ cells%: 78.24% ± 10.18% vs. 94.16% ± 4.20%, *p* = .0120, Figure 5F; IFN‐γ^+^ cells%: 0.44% ± 0.28% vs. 3.19% ± 1.59%, *p* = .0052, Figure 5G). Compared to CD161^−^CD56^+^ NK cells, the expression proportion of FasL on CD161^+^CD56^+^ NK cells significantly decreased (37.32% ± 8.69% vs. 64.04% ± 12.55%, *p* = .0045, Figure 5E). In addition, we also found that the expression proportion of granzyme B and perforin in CD161^+^CD56^+^ NK cells was lower than that on CD161^−^CD56^+^ NK cells (granzyme B^+^ cells%:86.86% ± 3.89% vs. 90.20% ± 2.38%, *p* = .1398, Figure 5H; perforin^+^ cells%:15.15% ± 7.68% vs. 15.85% ± 8.30%, *p* = .8939, Figure 5I), but there was no statistical difference. These results indicated that CD161 in peripheral blood of pSS patients inhibited the function of CD56^+^ NK cells.

## 4 DISCUSSION

pSS is a systemic autoimmune disease. In addition to dry symptoms caused by gland dysfunction, there are also extra‐glandular manifestations, which can be life‐threatening in severe cases, at the same time, these symptoms such as dryness, fatigue, and pain often adversely affect the quality of life in patients.^16^ At present, the therapy of pSS is mainly based on clinical experience and symptomatic treatment, such as alleviating dryness and applying total glucosides of paeony, hydroxychloroquine, and leflunomide to regulate immunity.^2^, ^17^ Biological therapy of pSS has a valuable application prospect, but its effectiveness and safety are still controversial. Therefore, it is essential to explore effective potential therapeutic targets for immunotherapy of pSS. NK cells are congenital lymphocytes, which can kill their target cells through perforin, granzyme, or death‐inducing receptors. At present, more and more evidence reveals the role of NK cells in autoimmune diseases such as systemic sclerosis (SSc), systemic lupus erythematosus (SLE), PBC and pSS.^6^, ^18^, ^19^, ^20^, ^21^ Although T cells and B cells are dominant in the pathogenesis of pSS, innate immune cells such as NK cells also play an important role in pSS.

In this study, we observed that the proportion of CD56^+^ NK cells in the peripheral blood of pSS patients (including active and inactive patients) was significantly lower than that of HCs. In recent years, it has also been reported that the frequency and absolute number of CD3^−^CD56^+^ NK cells in peripheral blood of pSS patients decreased significantly, and the ratio of CD56^bright^ NK to CD56^dim^ NK in peripheral blood may have relatively specific diagnostic value for pSS.^6^ It was speculated that the reduction of NK cells in the peripheral blood of pSS patients may be due to the augmented homing of cytotoxic cells to exocrine glands, which trigger and maintain tissue inflammation by producing Th1 cytokines and cytotoxic mediators.^6^ Another study found that the absolute number of NK cells in pSS patients with renal tubular acidosis (RTA) was significantly lower than that in patients without RTA.^4^ The above studies indicate that NK cells are involved in the disease development of pSS. Similarly, previous studies found that NK cells in peripheral circulation decreased in SSc, especially in patients with organ involvement, and speculated that the decrease of NK cell proportion in peripheral blood may be due to the infiltration of NK cells into the involved tissues.^22^ However, the reasons for the decrease of NK cells in pSS and the mechanism of NK cells affecting the development of pSS diseases need further study.

CD161 is a type C lectin‐like type II transmembrane protein, which is mainly expressed on the surface of most NK cells and circulating memory T cells.^23^, ^24^ Human CD161 binds to its ligand LLT1, and inhibits the activity and function of NK cells, but it is still controversial whether it inhibits or activates T cells.^25^ In pSS patients, there are few research on CD161. There were two studies on CD161 expression on T cells in peripheral blood of pSS and its clinical relevance with diseases. Zhao et al. found that the expression of CD161 on CD4^+^ T cells of pSS patients was higher than that in HCs, and the retinoic acid receptor‐related orphan nuclear receptor‐γ frequency on CD161^+^CD4^+^ T cells in peripheral blood increased, which was positively correlated with auto‐antibodies and hypergammaglobulinemia.^11^ Another study reported that compared with HCs, CD4^+^CD25^+^CD161^+^ T cell subsets significantly increased in the peripheral blood of pSS patients, and the proportion of IL‐17‐producing cells in CD161^+^ T cell was higher than that in CD161^−^ T cell, and CD4^+^CD161^+^ T cells in peripheral circulation were related to the activity and severity of pSS disease.^12^ These studies indicated that CD161 played an important role in the pathogenesis of pSS and may be a potential therapeutic target for pSS.

In this study, we found that the proportion of CD161 on the surface of CD56^+^ NK cells in peripheral blood decreased significantly compared with HCs. Similar results were also reflected in SLE. Two studies reported that the expression of CD161 decreased on the surface of NK cells in the peripheral circulation of SLE patients, and suggested that CD161^+^ NK cells were involved in the pathogenesis of SLE.^26^, ^27^ The receptors on the surface of NK cells, including activated receptors and inhibitory receptors, regulate the function of NK cells through balancing signal transmission. CD161, as an inhibitory receptor on the surface of NK cells, inhibits the transmission of cytotoxic functional signals of NK cells.^9^ Our study also observed that compared to CD161^−^CD56^+^ NK cells, the expression proportion of surface‐activated marker CD86 and HLA‐DR on CD161^+^CD56^+^ NK cells were significantly decreased, and the expression proportion of proliferation marker Ki67, apoptosis‐inducing molecule FasL, and the pro‐inflammatory cytokines TNF‐α and IFN‐γ significantly decreased. Therefore, we speculated that the decrease of CD161 expression on NK cells in peripheral blood of pSS patients weakened the inhibition function of NK cells, which led to the enhancement of cytotoxicity and the increased release of cytokine. Likewise, it was reported that the frequency of circulating CD56^+^CD161^+^ NK Cells decreased in human visceral leishmaniasis.^28^ All the above studies reflected that CD161 involved in the pathogenesis of autoimmune diseases and infectious diseases by mediating the function of NK cells.

We further observed that the proportion of CD161^+^CD56^+^ NK cells was associated with the clinical characteristics and laboratory parameters in pSS. The CD161^+^CD56^+^ NK cells proportion was significantly lower in pSS patients with decayed tooth, fatigue, arthralgia, skin involvement, PBC, and ILD than that in patients without above features. Furthermore, we found that the proportion of CD161^+^CD56^+^ NK cells in peripheral blood of active patients (ESSDAI > 5) reduced obviously compared with that in inactive pSS patients. Further clinical correlation analysis showed that the proportion of CD161^+^CD56^+^ NK cells was negatively correlated with disease activity and severity of pSS. These results suggested that the decrease of CD161^+^CD56^+^ NK cells may contribute to the progression of pSS. Lenart et al. found that activation of the LLT1‐CD161 axis can inhibit granzyme B and IFN‐γ production by NK cells and hamper the function of NK cells.^29^ CD161 is expressed in the early stage of NK cell development, and in the peripheral circulation, the crosslinking of CD161 leads to upregulate the expression of IFN‐γ and inhibits the cytotoxicity of NK cells.^10^, ^30^ Another study showed that CD161 on NK cells combined with its ligand on target cells and inhibited NK cytotoxicity by activating acidic sphingomyelinase.^31^ There was a tendency that granzyme B and perforin on CD161^−^CD56^+^ NK cells increased compared to those on CD161^+^CD56^+^ NK cells in our study, although there was no statistical significance. Thus, we speculated that the decrease of CD161 may affect the function of CD56^+^ NK cells through some mechanism, leading to enhanced cytotoxicity and increased secretion of inflammatory cytokines, and aggravating the progress of pSS disease.

One of the features of pSS is the production of auto‐antibodies and the increase of immunoglobulins in patients after the over‐activation of B cells.^32^ In our study, we also found that the proportion of CD161^+^CD56^+^ NK cells in peripheral circulation decreased significantly in pSS patients with anti‐SSA/Ro60 positive, anti‐SSB positive and high IgG. It has been shown that cytokines produced by NK cells, such as IFN‐γ can promote the activation of B cells and enhance the production of immunoglobulin.^33^ Rosen DB et al. reported that CD161 interacted with LLT1 expressed on activated B cells, regulating the crosstalk between NK cells and B cells.^10^ Early studies have also confirmed that NK cells can enhance the proliferation of B cells.^34^ Another research showed that human invariant NKT cells could directly help autologous B lymphocytes, induce the proliferation of naive and memory B cells, produce immunoglobulin and antibodies in vitro.^35^ Therefore, we speculated that CD161, as an inhibitory receptor of NK cells, decreased on the surface of CD56^+^ NK cells in pSS patients, which weakened the inhibition on NK cell function, and led to the increased secretion of cytokines such as IFN‐γ, promoted the activation and proliferation of B cells, and produced more auto‐antibodies and immunoglobulins in pSS patients.

However, there still exist some limitations in our research. According to the expression of CD56, human NK cells can be divided into CD56^bright^ and CD56^dim^ subsets. Our study did not deeply analyze the difference in CD161 expression on CD56^bright^ and CD56^dim^ subsets. On the other hand, this research was a cross‐sectional and observational study, and the number of participants recruited was small. We only analyzed the clinical correlation between the proportion of CD161^+^CD56^+^ NK cells and pSS, and it was not clear how CD161 mediated the function of NK cells to participate in the pathogenesis of pSS. It needs further study in the later stage. Finally, it is generally believed that CD56^+^ NK cell subsets in salivary glands of pSS patients are more appropriate to reflect the lesions in glands, but this study lacks the histopathological verification of target tissues such as salivary gland tissues. Next, we will further explore how CD161 mediates the function of NK cells involved in the pathogenesis of pSS from the above aspects.

## 5 CONCLUSION

In conclusion, in this study, we revealed that the proportion of CD56^+^ NK cells and CD161 on CD56^+^ NK cells in peripheral blood of pSS patients significantly decreased compared to HCs. The proportion of CD161^+^CD56^+^ NK cells was significantly correlated with the clinical features and laboratory parameters including auto‐antibodies and immunoglobulins in pSS patients, and negatively associated with disease activity and severity of pSS. The ROC curve showed that CD161^+^CD56^+^ NK cells had certain reference value for the diagnosis of pSS. Besides, CD161expression inhibited the function of CD56^+^ NK cells in peripheral blood of pSS patients. In short, our findings suggest that CD161^+^CD56^+^ NK cells may influence the progression of pSS and serve as a biomarker of disease activity and potential targets for therapy of pSS.

## AUTHOR CONTRIBUTIONS

**Ping Zhao**: Data curation; funding acquisition; investigation; writing—original draft; writing—review and editing. **Yanhong Yang**: Data curation; investigation; writing—review and editing. **Saizhe Song**: Data curation; investigation. **Wei Cheng**: Data curation; investigation. **Cheng Peng**: Data curation; investigation. **Xin Chang**: Conceptualization; funding acquisition; methodology; resources. **Jian Wu**: Conceptualization; funding acquisition; resources; supervision. **Cuiping Liu**: Conceptualization; data curation; funding acquisition; resources; writing—review and editing.

## CONFLICT OF INTEREST STATEMENT

The authors declare no conflict of interest.

## Supporting information

Supporting information.

## Data Availability

The data sets generated during and/or analyzed during the current study are available from the corresponding author on reasonable request.
