# Supplementary material for: The proportion of CD161 on CD56+ NK cells in peripheral circulation associates with clinical features and disease activity of primary Sjögren's syndrome
Source: Immun Inflamm Dis. 2024 Apr 5;12(4):e1244. doi: 10.1002/iid3.1244 (PMC10996382; doi:10.1002/iid3.1244)
Supplement: Supplementary file 1 — Supporting information. [file IID3-12-e1244-s001.docx]

Supplement Fig1

A


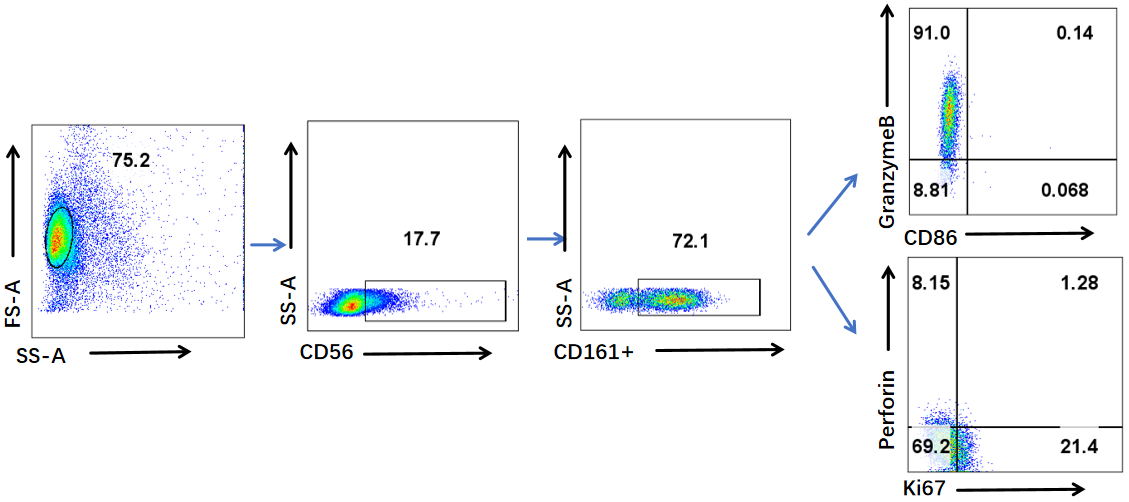


B


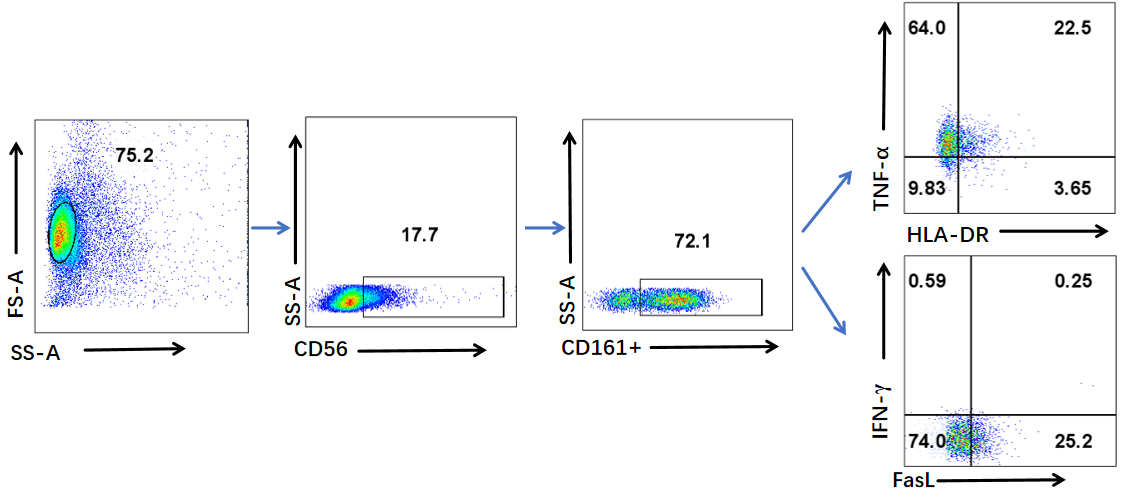


C


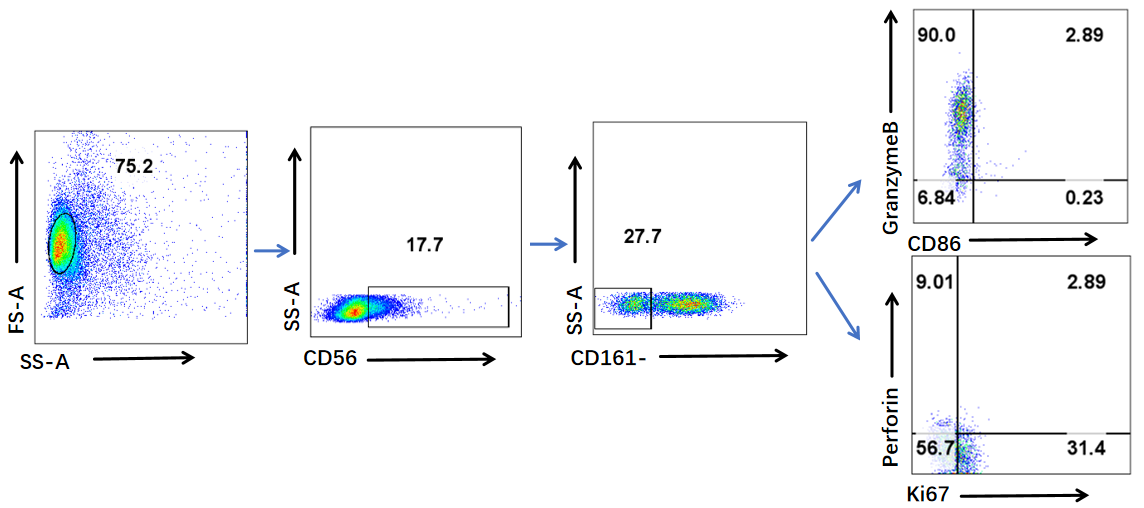


D


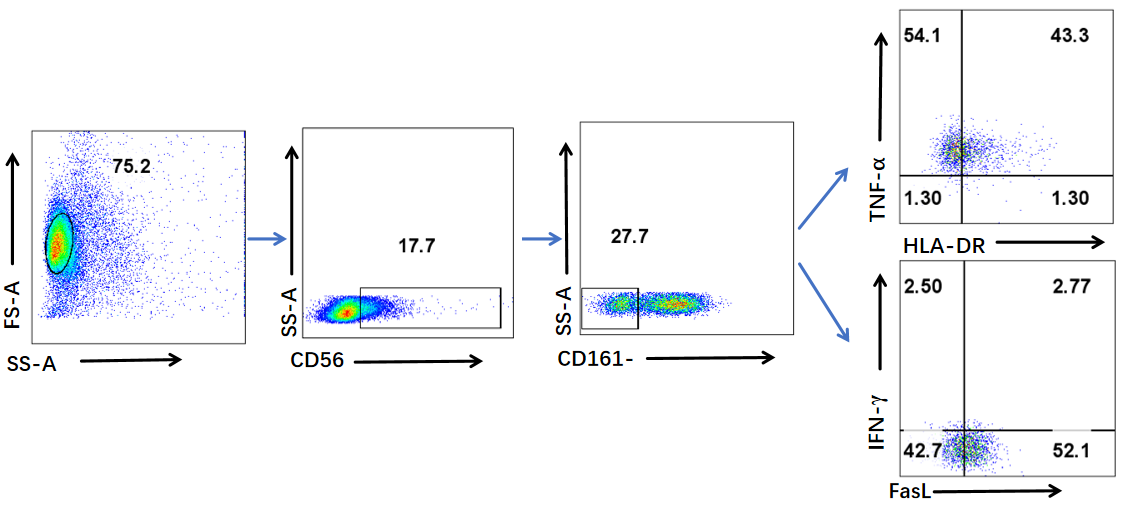


A.The gating strategies for CD86,granzyme B, perforin and Ki67 on CD161+CD56+NK cells .

B.The gating strategies for HLA-DR, TNF-α , IFN-γ and FasL on CD161+CD56+NK cells .

C.The gating strategies for CD86,granzyme B, perforin and Ki67 on CD161-CD56+NK cells.

D.The gating strategies for HLA-DR, TNF-α , IFN-γ and FasL on CD161-CD56+NK cells.
